# Supplementary material for: Aromatic acid metabolism in Methylobacterium extorquens reveals interplay between methylotrophic and heterotrophic pathways
Source: Appl Environ Microbiol. 2025 Sep 3;91(10):e00761-25. doi: 10.1128/aem.00761-25 (PMC12542667; doi:10.1128/aem.00761-25)
Supplement: Supplemental material — Figures S1 to S3; Table S1. [file aem.00761-25-s0001.docx]

**Supplementary Information**

**Figure S1.** Final yields of *M. extorquens* SLI 505 (“M. ext”, black) and *P. putida* KT2440 (“P. put”, white) on 5 and 12 mM vanillic acid. Error bars indicate standard deviation of 2-3 replicates.


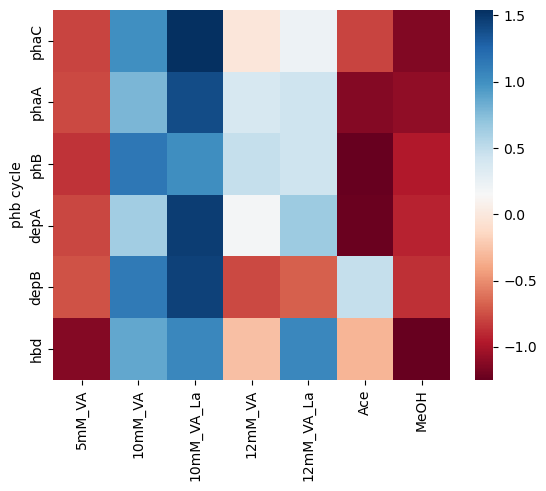

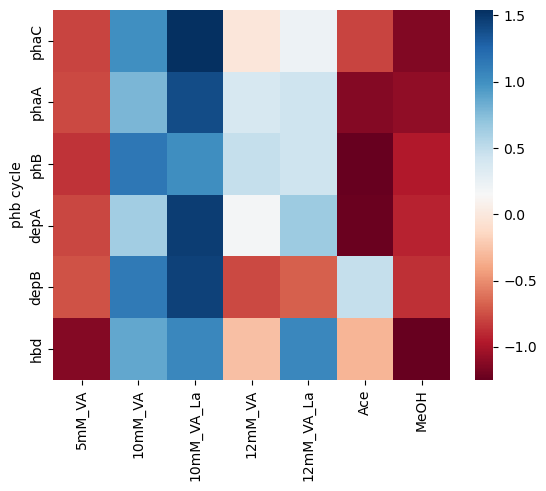


**PHB cycle genes**


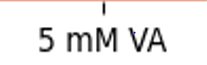

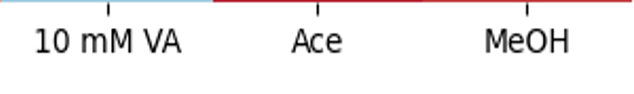

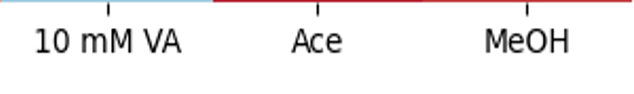

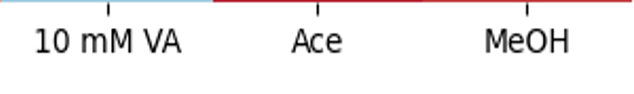


**C**

**A**


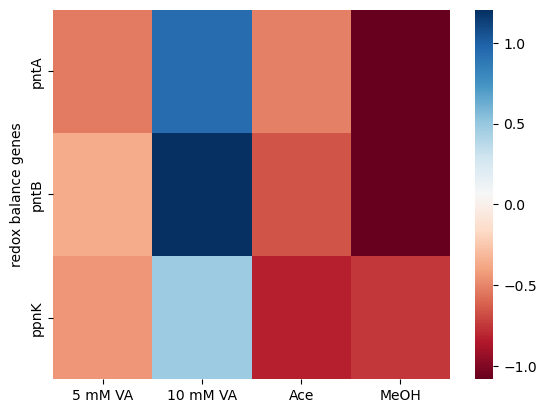


**redox balance genes**

**A**

**A**

**B**

**A**

**Figure S2.** Evidence for NADPH as an important currency metabolite during growth on vanillic acid. Normalized expression levels of methylotrophic and heterotrophic genes during growth on “low” (5 mM) and “high” (10 mM) concentrations of vanillic acid, 10 mM acetate, and 50 mM methanol. Expression profiles on methanol serve as a comparison for methylotrophic growth; expression profiles on acetate serve as a comparison for heterotrophic growth. Colors and corresponding color scale bar on the right of each graph indicates Z-score-corrected expression for comparisons of each gene across the four substrate conditions. **A.** polydhydroxybutyrate (PHB) cycle genes; **B.** redox balance genes. **C.** Nile Blue A staining and normalized fluorescent measurements of *M. extorquens* SLI 505 in low vanillic acid (5 mM VA), high vanillic acid (10 mM VA), and 50 mM methanol as a relative measurement of polyhydroxybutyrate concentrations. Averages of three biological replicates are reported. Error bars represent standard deviation

**Figure S3.** Propidium iodide staining of *M. extorquens* SLI 505 as a measurement of membrane permeability. *M. extorquens* SLI 505 was grown in 15 mM succinate (“succinate”), 50 mM methanol (“methanol”), 10 mM acetate (“acetate”), 5 mM vanillic acid (“5 mM VA”), 10 mM vanillic acid (“10 mM VA”), and 13 mM vanillic acid (“13 mM VA”) and stained with propidium iodide, a dye that fluoresces at 535/617 nm upon binding of DNA and can act as a proxy for measuring membrane permeability. Averages of two biological replicates and three technical replicates of samples corrected for background fluorescence of unstained cells are reported. Error bars represent standard deviation

**Table S1.** Strains and plasmids used in this study

| **Strain or Plasmid** | **Description** | **Reference** |
| --- | --- | --- |
| **Strains** |  |  |
| *Escherichia coli* 10β | Electrocompetent cloning strain | Invitrogen |
| *E. coli* S17-1 | Conjugating donor strain | New England Biolabs |
| *Methylobacterium extorquens* SLI 505 | Wild-type | 36 |
| *M. extorquens* SLI 505 Δ*ftfL* | Δ*ftfL* | This study |
| *M. extorquens* SLI 505 Δ*mptG* | Δ*mptG* | This study |
| **Plasmids** |  |  |
| pCM433KanT | SacB allelic exchange backbone; Km^R^ | 56 |
| pAG1 | SacB allelic exchange *ftfL* donor; Km^R^ | This study |
| pAG24 | SacB allelic exchange *mptG* donor; Km^R^ | This study |
